# Validation of a series of walking and stepping tests to predict maximal oxygen consumption in adults aged 18–79 years

**DOI:** 10.1371/journal.pone.0264110

**Published:** 2022-02-25

**Authors:** Taylor W. Rowley, Chris Cho, Ann M. Swartz, Young Cho, Scott J. Strath

**Affiliations:** 1 Department of Kinesiology, Saginaw Valley State University, University Center, MI, United States of America; 2 Department of Kinesiology, University of Wisconsin-Milwaukee, Milwaukee, WI, United States of America; 3 Zilber School of Public Health, University of Wisconsin-Milwaukee, Milwaukee, WI, United States of America; Universidade Estadual Paulista Julio de Mesquita Filho - Campus de Bauru, BRAZIL

## Abstract

**Introduction:**

Field tests to estimate maximal oxygen consumption (VO_2max_) are an alternative to traditional exercise testing methods. Published field tests and their accompanying estimation equations account for up to 80% of the variance in VO_2max_ with an error rate of ~4.5 ml^.^kg^-1.^min^-1^. These tests are limited to very specific age-range populations. The purpose of this study was to create and validate a series of easily administered walking and stepping field equations to predict VO_2max_ across a range of healthy 18-79-year-old adults.

**Methods:**

One-hundred-fifty-seven adults completed a graded maximal exercise test to assess VO_2max_. Five separate walking and three separate stepping tests of varying durations, number of stages, and intensities were completed. VO_2max_ estimation equations were created using hierarchal multiple regression. Covariates including age, sex, body mass, resting heart rate, distance walked, gait speed, stepping cadence, and recovery heart rate were entered into each model using a stepwise approach. Each full model created had the same base model consisting of age, sex, and body mass. Validity of each model was assessed using a Jackknife cross-validation analysis, and percent bias and root mean square error (RMSE) were calculated.

**Results:**

Base models accounted for ~72% of the total variance of VO_2max_. Full model variance ranged from ~79–83% and bias was minimal (<±1.0%) across models. RMSE for all models were approximately 4.5 ml^.^kg^-1.^min^-1^. Stepping tests performed better than walking tests by explaining ~2.5% more of the variance and displayed smaller RMSE.

**Conclusion:**

All eight models accounted for a large percentage of VO_2max_ variance (~81%) with a RMSE of ~4.5 ml^.^kg^-1.^min^-1^. The variance and level of error of models examined highlight good group mean prediction with greater error expected at the individual level. All the models perform similarly across a broad age range, highlighting flexibility in application of these tests to a more general population.

## Introduction

Maximal oxygen consumption (VO_2max_) is a key indicator of health and cardiorespiratory fitness [1] and is considered a “clinical vital sign” and strong predictor of mortality [2]. The traditional, gold standard method to assess VO_2max_ is open circuit spirometry in conjunction with a graded exercise test (GXT) to volitional fatigue. Open circuit spirometry, a method of indirect calorimetry, requires the use of a computerized metabolic measurement system to analyze expired gasses to determine oxygen utilization [1]. A standard GXT protocol, typically performed on a treadmill or cycle ergometer, incrementally increases exercise intensity until the participant achieves VO_2max_ [3]. Despite valuable information obtained from VO_2max_ testing, it is not always feasible in certain settings. The cost of the equipment required to complete such tests is high, and testing requires trained professionals, often making this form of testing inaccessible to the general public. Economic factors aside, VO_2max_ testing is not always a safe option for certain populations [1], such as the elderly who are at a higher risk for falling or those with an increased risk of experiencing an adverse cardiac event during vigorous exercise.

Submaximal VO_2_ testing to predict VO_2max_ is an alternative to traditional maximal testing without requiring the participant to work to a maximal intensity [1]. Two popular submaximal modalities are the treadmill and cycle ergometer [4–8]. Similar to maximal exercise testing, the cost associated with submaximal VO_2_ testing can be high and requires specialized equipment and trained personnel. Submaximal *field testing*, which involves simple equipment and measures (e.g. distance wheel, heart rate monitor), is another alternative to maximal exercise testing. Traditionally, these alternative, low cost options include over-ground walking/running [9–11] or stepping tests [7, 12, 13]. These tests can provide a safe testing alternative for high risk populations and can be easily administered in the field or clinical setting with little expense to estimate VO_2max_.

Ease of delivery and physical burden of a test are only two components to consider when selecting a field test to estimate VO_2max_. How well a field test prediction equation estimates VO_2max_, as determined through methodological validation research, and what population(s) the test is designed for are also important factors to consider. Explained variance and error of the estimate reported in the literature fluctuates among submaximal field tests predicting VO_2max_, with the highest performing prediction equations reporting in the region of 80% of the shared variance and an error of approximately 4.5 ml^.^kg^-1.^min^-1^ [8, 10]. Unfortunately, a limitation within the current body of literature is a lack of consistency in validation and reporting efforts [8]. Additionally, many of the published field tests tend to target homogenous groups of recreationally active young adults [6, 12] or adults with a narrow age range [10], with few studies developing and comparing field tests across a broad age range [13, 14]. Further, the modalities of these tests may be deemed inappropriate for certain populations, limiting their application to a broad, generalized population. Thus, there is a scientific need to examine the precision and accuracy of easily administered, low cost, submaximal field tests that transcend a wide age range. Accordingly, the purpose of this study was to determine the validity of various walking and stepping tests to predict VO_2max_ among a broad age-range of adults.

## Materials and methods

### Participants and study overview

This study had a cross-sectional design that spanned three days and two different settings. Day one of testing took place within a university laboratory on a large, midwestern campus. There, participants completed demographic, anthropometric, and VO_2max_ assessments, using the equipment and techniques outlined under the measures section. Days two and three took place at a separate, on-campus gymnasium with a climate controlled environment and a 200-meter indoor track. These testing days comprised of different walking and stepping exercise tests. One hundred and sixty-two individuals were recruited based on the following inclusion criteria: a.) age between 18–79 years old; b.) ambulatory (i.e. free of any walking limitations, such as use of an assistive device or amputation); c.) able to walk on a treadmill; and d.) healthy as determined by a physical examination within the past three years. Individuals were excluded if they: a.) had a diagnosis of a cardiovascular, metabolic, or pulmonary condition; b.) were pregnant or nursing; and c.) had a history of severe arthritis or other orthopedic conditions. Participants were recruited via telephone, flyers, and word of mouth from a large, metropolitan area and surrounding communities. This study was approved by the University of Wisconsin-Milwaukee Institutional Review Board, #08.298.

Written informed consent from the participants was obtained prior to enrollment to the study.

### Measures

#### Demographic and anthropometric assessment

Participants completed a health history questionnaire that assessed current health status and family health history. Height was measured to the nearest quarter of an inch using a stadiometer (Detecto, Webb City, MO, USA) and weight was measured to the nearest quarter of a pound using a calibrated physician’s scale (Detecto, Webb City, MO, USA), with which body mass index (BMI) was calculated. Resting blood pressure and heart rate were assessed using auscultation and palpitation, respectively, following standard procedures [15].

#### Maximal exercise test

A modified Balke treadmill protocol [1] was used to measure VO_2max_. Participants were fitted with a 3-way, non-rebreathing mouthpiece, nose clip, and head support (Hans-Rudolph) that were connected to a metabolic cart using a tube (TrueOne 2400, ParvoMedics, Sandy, UT, USA) to assess expired gas. Measurement of oxygen consumption through expired gasses using this metabolic cart has been previously validated against the traditional Douglas bag method. Specifically, excellent accuracy and precision was reported for gas exchange variables, and VO_2_ was found to differ by [0.018] l/min [4]. Heart rate and electrical activity were monitored using a 12-lead EKG (Case System, GE Healthcare, USA). Volitional fatigue or the following criteria had to be met to be considered a maximal exercise test: a plateau <2.1 ml/kg/min between two stages, a respiratory exchange ratio of 1.1 or greater, and a heart rate within 10 bpm of age-predicted maximal heart rate (220-age) [16].

#### Field tests

During the field tests, participants were fitted with a heart rate monitor (Polar, Polar Electro Inc., Bethpage, NY, USA) to measure recovery heart rate. All tests were separated by a minimum of 5-minutes of seated recovery. Additional time was given to the participant as they deemed it necessary. Heart rate returning back to baseline prior to each new test being started was used as a further marker of sufficient rest being obtained between tests administered. This was consistent for each field test.

#### Walking tests

Participants completed a series of over-ground walking tests (Table 1). Total distances (m) for single stage tests and individual-stage distance for ramped-intensity, multi-stage tests were measured using a Pittsburgh brand 10,000 ft/m distance wheel. Walking speed (m^.^s^-1^) was calculated by dividing distance with time and was recorded for single stage tests and individual stages for ramped-intensity protocol tests. Walking speeds were selected for ease of administration.

**Table 1:** Description of the walking tests.

Test #	Name	Description
1	Walk at 1 or 1.5 mph	5-minute walk to a cadence of 60 bpm
2	2-minute walk*	Cover as much ground as possible within the time frame
3	6-minute walk*	Stage 1: <
	(3-minute stages)	Stage 2: >
4	6-minute walk*	Stage 1: <
	(2-minute stages)	Stage 2: =
		Stage 3: >
5	9-minute walk*	Stage 1: <
	(3-minute stages)	Stage 2: =
		Stage 3: >

Summary of walking tests, including duration, number of stages, and walking speed.

*Walking speeds were self-selected for these tests. < slower than normal speed, = normal speed, > faster than normal speed.

Depending on the protocol (tests 3–5), participants were instructed to walk at a self-selected slower than normal, normal, and/or faster than normal walking speed. These walking speeds were self-determined. Additionally, the progressive nature of these walking tests emulates traditional graded exercise tests. Recovery heart rate was recorded at 30-second time points for two-minutes after each test.

#### Step tests

Test duration, stages per test, and stepping cadence were selected to mimic the progressive nature of traditional graded exercise tests (Table 2). Step height was selected to mimic traditional step height (e.g. on a flight of stairs) and two different heights were selected to further modify intensity levels. Stepping cadence was assigned based on age (Table 3) with the older age group(s) starting at a lighter intensity than the younger age group(s), to ensure that the test remained submaximal. Recovery heart rate was recorded at 30-second time points for two-minutes after each test.

**Table 2:** Description of the step tests.

Test #	Name	Description
6	6-minute step test	Three-minute stages
	(6-inch step)	Cadence increased after Stage 1
7	9-minute step test	Three-minute stages
	(6-inch step)	Cadence increased after Stage 1 and Stage 2
8	9-minute step test	Three-minute stages
	(8-inch step)	Cadence increased after Stage 1 and Stage 2

Summary of each step test. Cadence was assigned based on age and stage of test (see Table 3).

**Table 3:** Stepping cadence.

<40 years old	40–60 years old	>60 years old	Test/Stage Used
20 s/min = 80 bpm	15 s/min = 60 bpm	10 s/min = 40 bpm	Test 6/stage 1
			Test 7/stage 1
			Test 8/stage 1
25 s/min = 100 bpm	20 s/min = 80 bpm	15 s/min = 60 bpm	Test 6/stage 2
			Test 7/stage 2
			Test 8/stage 2
30 s/min = 120 bpm	25 s/min = 100 bpm	20 s/min = 80 bpm	Test 7/stage 3
			Test 8/stage 3

Stepping cadence was assigned based on age and stage of test.

s/min = steps per minute; bpm = beats per minute.

### Statistical analysis

Statistical analysis was completed in SPSS Version 22. Hierarchal regression analysis (using stepwise selection) was used to build models to predict VO_2max_. The base model for each equation consisted of age (years), sex (male = 1, female = 0), and body mass (kg), and was entered as the first step of the model. Resting heart rate (bpm) and recovery heart rate (bpm) variables were entered into each model. Walking distance (m) and walking speed (m^.^s^-1^) were entered into walking test models, and step cadence (bpm) and step height (in) were entered into step test models. For ramped protocol walking tests, individual-stage distance, individual-stage speed, total distance, and average speed were included when building the equations. Variables that significantly predicted VO_2max_ were kept in the model, while variables that did not significantly predict VO_2max_ were excluded. Main effects were only considered due to sample size limitations. The resulting model from hierarchical and selection process were tested for multicollinearity using variance inflation factor (VIF). Variables identified with a high VIF (>1.0) were removed from the model. Explained variance (R^2^), adjusted R^2^ (R^2^_adj_), and root mean square error (RMSE) were generated for each model.

Each regression equation was then cross-validated using the Jackknife analysis (leave one subject out) method [17] using SAS Version 9.4. Bias and RMSE were created for each test predicting VO_2max._ Bland-Altman plots [18] and 95% limits of agreement (LoA, SD of the differences 1.96) were created and a t-test for differences between measured and predicted VO_2max_ values was assessed. Significance for all tests was set at *p*<0.05.

## Results

Five of the 162 participants recruited did not qualify for the study. Of the final 157 participants, two-thirds of the sample was female (66%) and the average age was 48.9 ± 17.4years (mean ± SD). Average measured VO_2max_ was 34.3 ± 10.1 ml^.^kg^-1.^min^-1^ and average BMI was 25.7 ± 4.3 kg^.^m^-2^. Participant characteristics broken down by sex are presented in Table 4.

**Table 4:** Participant characteristics.

	Female (n = 83)	Male (n = 74)	All (n = 157)
Age (yrs)	50.4 ± 16.5	47.2 ± 18.1	48.9 ± 17.4
Height (cm)	164.7 ± 5.9	178.3 ± 7.1	171.1 ± 9.4
Body Mass (kg)	67.5 ± 12.9	85.4 ± 14.9	75.9 ± 16.5
BMI (kg^.^m^-2^)	24.9 ± 4.5	26.8 ± 3.9	25.7 ± 4.3
Resting Heart Rate (bpm)	60.0 ± 9.0	60.8 ± 8.8	60.3 ± 8.9
VO_2max_ (ml^.^kg^-1.^min^-1^)	31.5 ± 9.7	37.5 ± 9.7	34.3 ± 10.1

Data presented as Mean ± SD.

Characteristics of participants within the total sample and of participants who had complete data.

### Base model

The base model for each regression equation included age (years), sex (male), and body mass (kg). While the specific values for the base model varied among tests, this model alone accounted for ~72% of the explained variance in VO_2max_ and the RMSE was approximately 5.45 ml^.^kg^-1.^min^-1^. Age and body mass had a negative relationship with VO_2max_ meaning that as age or body mass increased, VO_2max_ decreased. Male sex, alternatively, was associated with a higher VO_2max_. This relationship was true across all base models, which are reported in Tables 5 and 6 for the walking and stepping equations, respectively.

**Table 5:** Regression equations for the walking tests.

		TEST 1		TEST 2		TEST 3		TEST 4		TEST 5	
		n = 149		n = 146		n = 147		n = 147		n = 149	
	Predictor	B	SE(B)	B	SE(B)	B	SE(B)	B	SE(B)	B	SE(B)
**Base Model**	*Constant*	**71.076**	2.592	**70.862**	2.610	**70.947**	2.610	**71.239**	2.603	**71.076**	2.592
	*Age*	**-0.398**	0.026	**-0.396**	1.066	**-0.399**	0.026	**-0.397**	0.026	**-0.398**	0.026
	*Male*	**10.163**	1.060	**10.198**	0.026	**10.208**	1.064	**10.082**	1.067	**10.163**	1.060
	*Body Mass*	**-0.290**	0.032	**-.289**	0.032	**-0.288**	0.032	**-0.291**	0.032	**-0.290**	0.032
	R^2^	0.717		0.717		0.720		0.717		0.717	
	R^2^ (Adjusted)	0.712		0.711		0.714		0.711		0.712	
	RMSE	5.456		5.466		5.458		5.469		5.456	
**Full Model**	*Constant*	**79.666**	4.270	**51.366**	4.202	**60.952**	4.257	**63.783**	4.042	**61.664**	4.285
	*Age*	**-0.387**	0.022	**-0.319**	0.023	**-0.347**	0.023	**-0.357**	0.023	**-0.340**	0.023
	*Male*	**8.869**	0.928	**6.681**	0.925	**7.756**	0.934	**8.075**	0.948	**8.045**	0.956
	*Body Mass*	**-0.249**	0.028	**-0.193**	0.027	**-0.211**	0.028	**-0.214**	0.029	**-0.204**	0.029
	*Gait Speed (Total)*	**11.128**	4.921	**11.657**	1.446						
	*Gait Speed (Normal)*							**10.478**	2.139	**10.756**	2.212
	*Gait Speed (>Normal)*					**11.029**	2.049				
	*Heart Rate Recovery (30 s)*	**-0.248**	0.036			**-0.169**	0.023	**-0.151**	0.023	**-0.151**	0.023
	*Heart Rate Recovery (60 s)*			**-0.158**	0.021						
	R^2^	0.797		0.829		0.811		0.800		0.802	
	R^2^ (Adjusted)	0.790		0.823		0.804		0.793		0.794	
	RMSE	4.656		4.282		4.517		4.627		4.603	

Individual regression results for the five, over ground walking tests. **Bolded** values are significant (*p*<0.05). B = Unstandardized beta; SE(B) = Standard Error for the unstandardized beta; RMSE = Root Mean Square Error.

Test 1: Walk at 1 or 1.5 mph (single stage), 5-minute walk, cadence = 60 bpm

Test 2: 2-minute walk (single stage), cover as much distance as possible

Test 3*: 6-minute walk (3-minute stages), stage 1: < walking speed, stage 2: > walking speed

Test 4*: 6-minute walk (2-minute stages), stage 1: < walking speed, stage 2: = walking speed, stage 3: > walking speed

Test 5*: 9-minute walk (3-minute stages), stage 1: < walking speed, stage 2: = walking speed, stage 3: > walking speed

*Self-selected walking speeds.

**Table 6:** Regression equations for the step tests.

		TEST 6		TEST 7		TEST 8	
		n = 148		n = 145		n = 141	
	Predictor	B	SE(B)	B	SE(B)	B	SE(B)
**Base Model**	*Constant*	84.722	2.573	70.995	2.566	70.445	2.641
	*Age*	-0.433	0.026	-0.397	0.026	-0.401	0.026
	*Male*	10.293	1.053	10.141	1.060	9.952	1.091
	*Body Mass*	-0.290	0.032	-0.290	0.032	-0.277	0.033
	R^2^	0.722		0.722		0.720	
	R^2^ (Adjusted)	0.716		0.716		0.713	
	RMSE	5.408		5.379		5.375	
**Full Model**	*Constant*	**84.722**	2.494	**83.841**	2.421	**84.569**	2.457
	*Age*	**-0.433**	0.021	**-0.446**	0.021	**-0.329**	0.06
	*Male*	**7.724**	0.869	**7.181**	0.886	**6.825**	0.886
	*Body Mass*	**-0.183**	0.027	**-0.178**	0.027	**-0.181**	0.027
	*Stepping Cadence*					**-2.696**	1.283
	*Heart Rate Recovery (30 s)*	**-0.211**	0.022	**-0.183**	0.019	**-0.168**	0.017
	R^2^	0.830		0.831		0.835	
	R^2^ (Adjusted)	0.825		0.826		0.830	
	RMSE (Adjusted)	4.257		4.21		4.138	

Individual regression results for the three stepping tests. **Bolded** values are significant (*p*<0.05). B = Unstandardized beta; SE(B) = Standard Error for the unstandardized beta; RMSE = Root Mean Square Error.

Test 6: 6-minute step test (3-minute stages), cadence^†^ increase after stage 1, 6-inch step

Test 7: 9-minute step test (3-minute stages), cadence^†^ increase after stage 1 and 2, 6-inch step

Test 8: 9-minute step test (3-minute stages), cadence^†^ increase after state 1 and 2, 8-inch step

^†^Cadence varied by age and test stage. Cadence was lower as age increased.

#### Full models

Models were constructed on a test-by-test basis. Estimation of VO_2max_ was strong across all prediction equations. The explained variance for the field test equation models varied from 79.7% to 83.5%, with Test 1 (the five-minute walking test) being the weakest predictor of VO_2max_. Test 8 (the three stage, nine-minute step test using an 8-inch step) was the strongest predictor of VO_2max._ Likewise, RMSE for these tests ranged from 4.138 ml^.^kg^-1.^min^-1^ to 4.656 ml^.^kg^-1.^min^-1^ for Test 8 and Test 1, respectively. By adding variables to the base models, the full models were able to account for approximately 10% more explained variance in VO_2max_.

#### Walking regression equations

Walking regression results are presented in Table 5. Gait speed and recovery heart rate were common predictors among the walking equations. Gait speed, when significant, had a positive relationship with VO_2max_, where a faster-selected gait speed was associated with a higher VO_2max_. For the tests with multiple stages (Test 3–5), slower than usual gait speed was never a significant predictor. Heart rate variables varied among the tests and included 30- or 60-second recovery heart rate. All heart rate variables had a negative relationship with VO_2max_.

#### Stepping regression equations

Stepping regression results are presented in Table 6. Thirty-second recovery heart rate was a significant predictor for each step test. Like the walking tests, heart rate variables were negatively related to VO_2max_. Test 8 performed better than any of the other tests (walking or stepping) for predicting VO_2max_ (R^2^ = 0.835, R^2^_adj_ = 0.830, and RMSE = 4.138 ml^.^kg^-1.^min^-1^).

### Jackknife validation results

Results of the jackknife validation revealed that bias was relatively small for each test, with each model reporting a bias well within ± 1%. Root mean square error ranged from 4.102 ml^.^kg^-1.^min^-1^ to 4.662 ml^.^kg^-1.^min^-1^, for Test 8 and Test 1, respectively. Jackknife results are presented in Table 7.

**Table 7:** Summary of Jackknife validation analysis.

	TEST 1		TEST 2		TEST 3		TEST 4		TEST 5		TEST 6		TEST 7		TEST 8	
	B	%BIAS	B	%BIAS	B	%BIAS	B	%BIAS	B	%BIAS	B	%BIAS	B	%BIAS	B	%BIAS
*Constant*	79.50	-1.15 E-03	51.50	9.30 E-04	60.92	-1.14 E-03	63.59	-1.32 E-03	61.42	-2.72 E-05	84.62	-6.75 E-04	83.78	-4.38 E-04	84.57	1.91 E-06
*Age*	-0.39	4.33 E-06	-0.32	8.85 E-07	-0.35	9.10 E-06	-0.36	4.55 E-06	-0.34	-2.29 E-05	-0.43	5.91 E-06	-0.45	6.39 E-06	-0.33	1.93 E-05
*Male*	8.85	-1.52 E-04	6.66	-1.19 E-04	7.73	-3.51 E-04	8.05	-1.66 E-04	8.02	-2.34 E-05	7.74	8.77 E-05	7.18	2.61 E-05	6.83	4.85 E-05
*Body Mass*	-0.25	2.26 E-06	-0.19	3.20 E-06	-0.21	4.66 E-06	-0.21	2.32 E-06	-0.20	-3.45 E-05	-0.18	-3.98 E-06	-0.18	-5.90 E-06	-0.18	-8.04 E-06
*Gait Speed (Total)*	11.28	1.03 E-03	11.59	-4.59 E-04												
*Gait Speed (Normal)*							10.53	3.74 E-04	10.83	4.56 E-05						
*Gait Speed (>Normal)*					11.01	-1.41 E-04										
*Stepping Cadence*															-2.74	-3.05 E-04
*Heart Rate Recovery (30 s)*	-0.25	2.65 E-06			-0.17	5.84 E-07	-0.15	5.28 E-06	-0.15	-1.03 E-05	-0.21	7.30 E-06	-0.18	5.87 E-06	-0.17	2.24 E-06
*Heart Rate Recovery (60 s)*			-0.16	-2.92 E-06												
R^2^	0.791	-4.21 E-05	0.824	-3.64 E-05	0.805	-4.89 E-05	0.794	-4.56 E-05	0.795	-5.33 E-05	0.825	-3.06 E-05	0.826	-3.23 E-05	0.834	-4.11 E-05
RMSE	4.662	4.06 E-05	4.287	3.80 E-05	4.526	3.74 E-05	4.640	8.22 E-05	4.613	1.33 E-05	4.259	9.08 E-05	4.223	9.58 E-05	4.102	1.04 E-04

All estimates presented are bias adjusted Jackknife estimates. Percent bias is calculated as $\frac{Original\;Estimate-Jackknife\;Estimate}{Jackknife\;Estimate}\times 100\%$.

Walking Test Key

Test 1: Walk at 1 or 1.5 mph (single stage), 5-minute walk, cadence = 60 bpm

Test 2: 2-minute walk (single stage), cover as much distance as possible

Test 3*: 6-minute walk (3-minute stages), stage 1: < walking speed, stage 2: > walking speed

Test 4*: 6-minute walk (2-minute stages), stage 1: < walking speed, stage 2: = walking speed, stage 3: > walking speed

Test 5*: 9-minute walk (3-minute stages), stage 1: < walking speed, stage 2: = walking speed, stage 3: > walking speed

*Self-selected walking speeds.

Step Test Key

Test 6: 6-minute step test (3-minute stages), cadence^†^ increase after stage 1, 6-inch step

Test 7: 9-minute step test (3-minute stages), cadence^†^ increase after stage 1 and 2, 6-inch step

Test 8: 9-minute step test (3-minute stages), cadence^†^ increase after state 1 and 2, 8-inch step

^†^Cadence varied by age and test stage. Cadence was lower as age increased.

**Fig 1:**
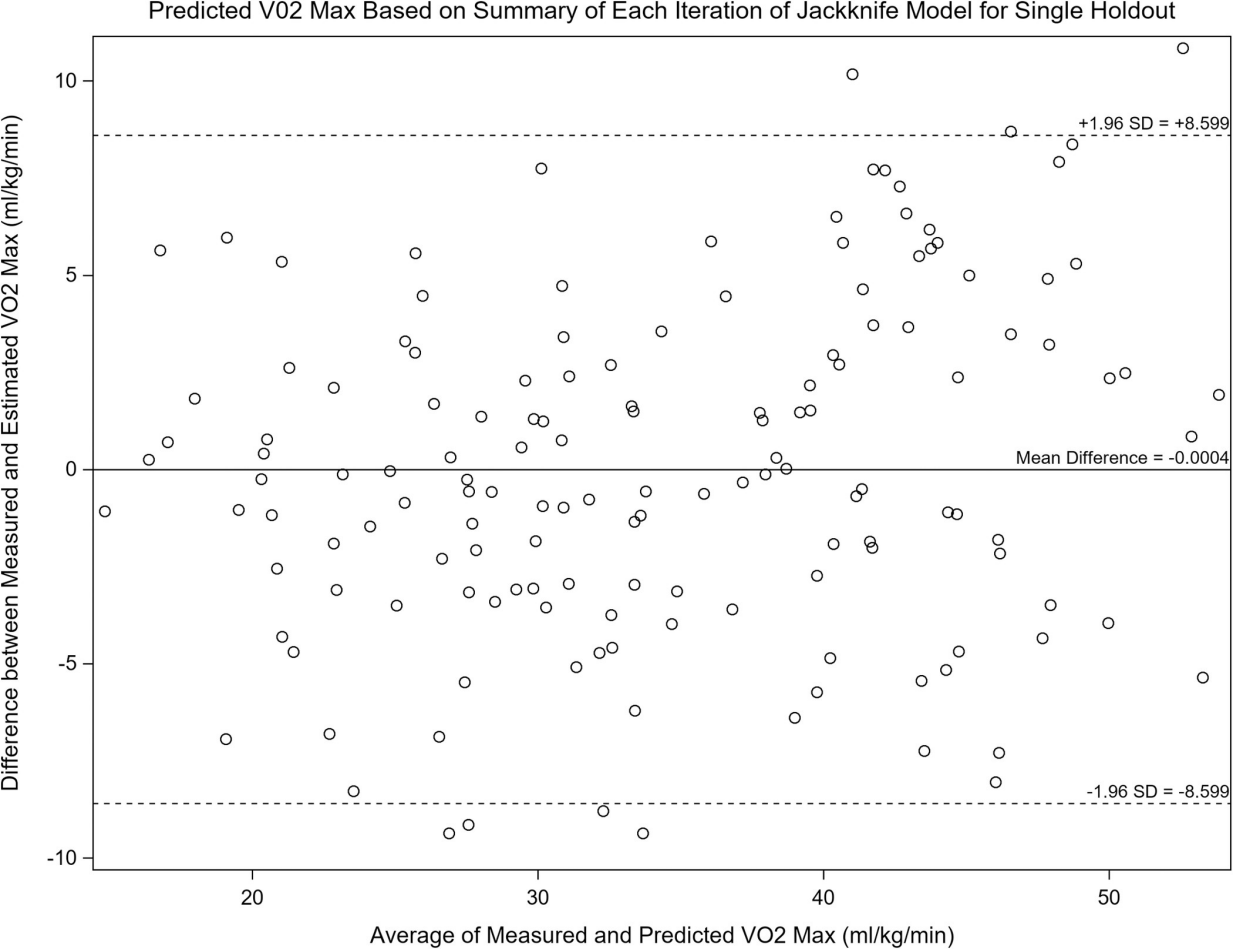
Bland-Altman plot for Test 2 (2-minute walking test). Figure shows mean error to be close to zero (-0.0004) and the limits of agreement are +/- 8.599 ml/kg/min. This indicates that there is minimal bias between the measured and predicted VO2max values.

**Fig 2:**
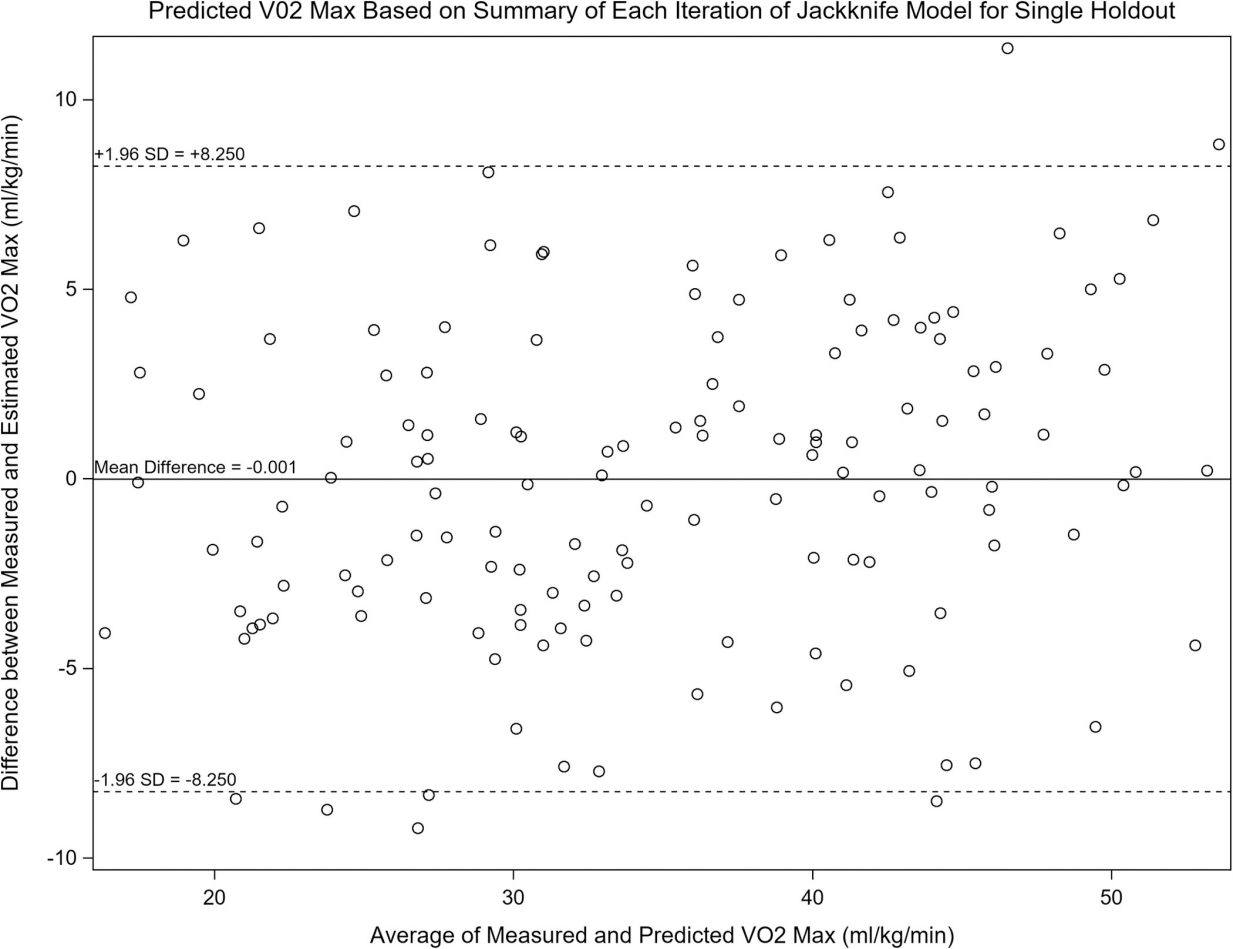
Bland-Altman plot for Test 8 (9-minute step test). Figure shows mean error to be close to zero (-0.001) the limits of agreement are +/- 8.250 ml/kg/min. This indicates that there is minimal bias between the measured and predicted VO2max values.

Of the walking tests, the model for Test 2 still accounted for the greatest explained variance in VO_2max_ with a Jackknife adjusted R^2^ of 0.824 and RMSE of 4.287 ml^.^kg^-1.^min^-1^, and bias of -0.0000421% and 0.0000406%, respectively. Of the stepping tests, the model for Test 8 accounted for the greatest explained variance in VO_2max_ with a Jackknife adjusted R^2^ of 0.834 and RMSE of 4.102 ml^.^kg^-1.^min^-1^, and bias of -0.0000411% and 0.000104%, respectively. Bland-Altman plots were created for Test 2 (Fig 1) and for Test 8 (Fig 2). Plots show mean error to be close to zero, and LoA of +8.599 to -8.599 ml/kg/min (t-test, -0.000445) for Test 2 and +8.250 to -8.250 ml/kg/min (t-test, -0.001) for Test 8. Both Figs 1 and 2 show that there is no systematic bias of the prediction noted across the sample.

## Discussion

The purpose of this study was to determine the validity of several easily administered walking and stepping field-tests to predict VO_2max_ across a broad age range. We found that among all eight tests examined, the 9-minute stepping test with three stages, using an 8-inch step yielded the highest bias-adjusted R^2^ (0.834) and lowest RMSE (4.102 ml^.^kg^-1.^min^-1^) while maintaining minimal bias, well within ±1%. Overall, the stepping tests outperformed the walking tests for predicting VO_2max_ by having the highest bias-adjusted R^2^ values and lowest RMSE. However, of the walking tests, a single stage, two-minute test to walk as far as possible yielded the highest bias-adjusted R^2^ (0.824) and lowest RMSE (4.287 ml^.^kg^-1.^min^-1^), also maintaining a minimal bias within ±1%.

Three popular field tests that are widely used are the Queen’s College Step Test [12], Cooper 12-minute run [9], and the one-mile walk test [10]. The Queen’s College Step Test is a 3-minute, single stage step test that requires participants to maintain a cadence of 22 steps/min as they step up and down from a 16.25-inch step and then manually measure and record recovery heart rate [12]. Despite being a single stage test, which makes the test itself shorter, a step height that is close to a foot and a half tall makes this test rigorous and concerns related to balance and fall risk need to be considered. Alternatively, the step tests presented in the current study are 6 and 8-inches tall, which is comparable to a standard step height.

Stepping tests can be difficult to administer at times, as they require the participant to maintain a certain cadence while stepping up and down. Benefits of walking and running tests is that the participant can self-regulate. For example, both the Cooper 12-minute run test and the one-mile walk test instruct participants to cover as much ground within the time frame and walk as quickly as possible to complete the mile, respectively [9, 10]. The simplest of the walking tests in the current study was a two-minute test that asked participants to cover as much ground as possible while still maintaining a walk. These simple instructions paired with a short duration make this test very easy to administer and highly achievable for most individuals. Further, as the participants are walking, it is possible to measure the distance as they go, unlike the Cooper 12-minute run where distance can be difficult to gauge depending on the location of the test.

The field tests in the current study performed well when predicting VO_2max_, accounting for approximately 80% of the explained variance and yielding RMSE of approximately 4.5 ml^.^kg^-1.^min^.-1^. The Queen’s College Step Test reports a low R^2^ value of 0.563 [12], which accounts for ~30% less of the explained variance of VO_2max_ than our highest performing step test. The Cooper 12-minute run and the one-mile walk test report explained variances for VO_2max_ of around 77% and 81%, respectively [9, 10]. The explained variance for both the one-mile walk and Cooper 12-minute test is similar, albeit lower than the explained variance we report within for our walking tests in the current study. McArdle et al., reports a standard error, however the units are in ml^.^min^-1^, making it difficult to compare error rates among tests [12]. Cooper did not report an error for the 12-minute run estimation equation [9], but the one-mile walk test reported an associated error of 5.0 ml^.^kg^-1.^min^-1^ [10] which is marginally higher than what we report with our current study findings. Error associated with an equation can impact the interpretation of a score. Too large of an error of the estimate can make it difficult to detect true change in a variable (i.e. VO_2max_), and thus smaller error is preferred.

Cross validation analysis showed that our tests yielded minimal bias, meaning that the estimated VO_2max_ values were very similar to the measured VO_2max_ values. Unfortunately, there is inconsistency within the literature regarding validation reporting efforts, including the three previously published field tests listed above [9, 10, 12]. Kline and colleagues did, however, perform a cross-validation analysis in a separate sample and reported a final, adjusted variance of ~77% (R^2^ = 77.4) and standard error of 4.4 ml^.^kg^-1.^min^-1^ [10]. Although the error is similar to the ones we report here, the explained variance is lower than we found in the current study.

Some considerations are warranted when utilizing any of the field tests we report on. First, when considering feasibility and safety, the 9-minute stepping test, using an 8-inch step might not be appropriate for elderly or frail populations. As there was minimal difference in equation performance between the 9-minute stepping test using a 6-inch step and the 6-minute stepping test using a 6-inch step (~1% in variance and ~.1 ml^.^kg^-1.^min^-1^ in error), the shorter duration test with the shorter step could be a safer more practical option. Still, any form of stepping test could still perpetuate the risk for falls. The two-minute over-ground walking test could be the best option for a quick estimation of VO_2max_ as it requires minimal equipment and is shorter in duration. Additionally, the instructions are simple (“cover as much ground as possible in two-minutes”), whereas the stepping tests require a ramped cadence protocol which could cause confusion. Compared to the stepping tests, the two-minute walking test accounts for a similar amount of variance in VO_2max_ as the stepping tests (~82%) and contains a similar level of error (~4.2 ml^.^kg^-1.^min^-1^).

This study is not without limitations. First, the sample size was relatively small, which limited the analysis to only include main effects. Future studies should aim for a larger sample to allow for the investigation of interactions to potentially strengthen the model(s) to better predict VO_2max_. Second, while these models are statistically sound, further investigation into the application of these measures should be investigated. In a clinical setting or as a baseline estimate, any of these tests should be acceptable for estimating VO_2max_. The testing environment should also be considered when administering these tests, as they were developed in a climate-controlled environment. Factors, such as temperature, humidity, and wind could impact test results, thus altering the reliability of the estimation. Further, these models were developed in healthy adults, thus these results are limited to that population. Finally, despite assessing how well our models performed compared to the traditional gold-standard of open circuit spirometry for assessing VO_2max_, we did not compare our models to previously validated field test, which may have been a beneficial comparison to make.

In conclusion, this study generated VO_2max_ estimation equations from eight different stepping and over-ground walking field tests. A jackknife cross-validation assessment followed the creation of each equation to provide information on bias of each equation. By incorporating this bias, which was small, each equation accounted for ~80% of the explained variance for predicting VO_2max_ with an error of ~4.5 ml^.^kg^-1.^min-^1^. These results highlight that reported tests perform well to estimate group mean VO_2max_ values, but larger error would be expected for a given individual as the Bland-Altman plots display errors of ±8–9 ml^.^kg^-1.^min^-1^. Compared to previously published field tests, the tests presented here are appropriate for a broad age range and are simple to administer, requiring minimal equipment.

## Supporting information

S1 Data (SAV)
